# Epstein-Barr Virus: An Infrequent Pathogen of Acute Undifferentiated Febrile Illness From a Tertiary Care Hospital in Southern India

**DOI:** 10.7759/cureus.18207

**Published:** 2021-09-23

**Authors:** Rahul Dhodapkar, Mugunthan M, Kalpana Thangavelu, Monika Sivaradjy, Kowsalya Veerappan, Anitha Gunalan

**Affiliations:** 1 Department of Microbiology, Jawaharlal Institute of Postgraduate Medical Education and Research, Puducherry, IND; 2 Department of Microbiology, Sanjay Gandhi Postgraduate Institute of Medical Sciences, Lucknow, IND

**Keywords:** acute undifferentiated febrile illness, viral capsid antigen, epstein-barr nuclear antigen, ebv serology, epstein-barr virus

## Abstract

Context: Acute undifferentiated febrile illness (AUFI) is characterized by a sudden onset of raised body temperature and is a common cause of hospital admission though not recognized as a disease state by the World Health Organization. Epstein-Barr virus (EBV) is reported to account for a significant occurrence of AUFI cases.

Aim: To know the role of EBV infection as a cause of acute undifferentiated febrile illness (AUFI).

Settings and design: We have used the combination of EBV serological assays to establish the role of the Epstein-Barr virus as the cause of acute undifferentiated febrile illness.

Methods and material: A total of 721 suspected cases of acute undifferentiated febrile illness which were tested negative for other common causes of acute febrile illness were selected for the study. Serum samples collected from these cases were tested for the presence of the EBV viral capsid antigen (VCA) IgM antibody. All positive serum samples were tested for the presence of EBV Epstein-Barr nuclear antigen (EBNA) IgG.

Statistical analysis used: Statistical analysis was performed with the help of Microsoft Excel software (Microsoft Corporation, Redmond, USA).

Results: Out of 721 suspected AUFI cases tested for EBV VCA IgM antibodies, 117 samples were positive and 604 were negative. All these 117 samples were tested for EBV EBNA IgG antibodies in which 88 were positive and 29 were negative. In our study, we found that around 4% (positive for VCA IgM and negative for EBNA IgG) of AUFI cases can be attributed to primary acute EBV infection.

Conclusions: EBV infection should be considered particularly in AUFI cases of less than five years of age even in those who do not meet the typical presentation of fever, lymphadenopathy and sore throat. Our study should help to raise awareness regarding the possibility of EBV infection particularly in AUFI cases. A high index of suspicion and timely diagnosis will definitely help clinicians to avoid a battery of investigations and misuse of antibiotics in cases of AUFI.

## Introduction

Acute undifferentiated febrile illness (AUFI) is characterized by a sudden onset of raised body temperature without an apparent aetiology for two weeks duration and is a most frequent cause of hospital admission though not considered as a serious illness by the World Health Organization (WHO). Infectious aetiologies of AUFI lead to significant distress and mortality among children worldwide [1]. In countries like us where meagre resources impede diagnosis, clinical treatment is seldom reinforced by the knowledge of the cardinal regional pathogens [2-3]. The diagnostic approach in AUFI includes a thorough history taking and frequent physical examinations [4]. Predominant patients develop symptoms such as low-grade fever with rash, arthralgia, headache and myalgia with no apparent site of infection requiring laboratory confirmation for clinical management [5-7]. Malaria, typhoid, dengue, typhus and acute bacterial infections such as pneumonia are the major causes in resource-poor countries. In our own experience, we could make out that around 20%-30% of AUFI cases remain undiagnosed. Epstein-Barr virus (EBV) infection is a significant cause of AUFI cases though asymptomatic mostly [8-9]. Symptomatic EBV infection is difficult to be differentiated from those of other febrile illnesses [10-12]. Thus, the extent to which EBV causes AUFI remains unexplored. Using serological parameters like viral capsid antigen (VCA) IgM and Epstein-Barr nuclear antigen 1 (EBNA-1) IgG, acute and past EBV infections in immunocompetent individuals can be differentiated [13-14]. The presence of VCA IgM in the absence of EBNA-1 IgG indicates acute infection. In our study, we have used the combination of these two serological assays to establish the role of the EBV as the cause of AUFI.

## Materials and methods

**Figure 1:**
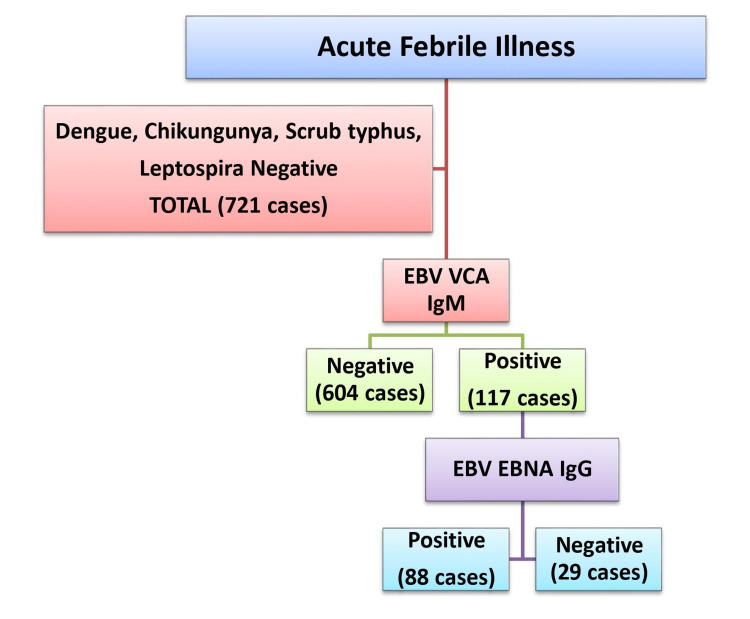
Flowchart of testing methodology followed for suspected AUFI cases AUFI: acute undifferentiated febrile illness, EBV: Epstein-Barr virus, EBNA: Epstein-Barr nuclear antigen, VCA: viral capsid antigen.

The aim of our study was to ascertain the role of EBV infection in AUFI patients who tested negative for all other typical microorganisms causing acute undifferentiated febrile illness. A total of 721 clinically suspected AUFI patients who were tested negative for malaria, arboviral (dengue, chikungunya), rickettsial (scrub typhus), leptospirosis and those with blood culture negative were included in the study (Figure 1). Serum samples collected from these suspected patients were tested for the appearance of EBV VCA IgM antibody by using Novalisa EBV VCA IgM enzyme-linked immunosorbent assay (ELISA) (NovaTec Immundiagnostica, Germany). Samples are considered positive if the absorbance value is higher than 11 Novalisa test units (NTU), and the absorbance value of less than 9 NTU is taken as negative. All positive serum samples were tested for the determination of EBV EBNA IgG by enzyme-linked fluorescence assay (ELFA) (Mini VIDAS, Biomerieux, France). Samples are considered positive if the test value is higher than 0.21 units, and the test value of less than 0.09 units is taken as negative. The Institutional Review Board approval was not obtained as the testing was performed with the archived samples preserved in our laboratory. Statistical analysis was performed with the help of Microsoft Excel 2007 (v12.0) (Microsoft Corporation Redmond, USA).

## Results

**Figure 2:**
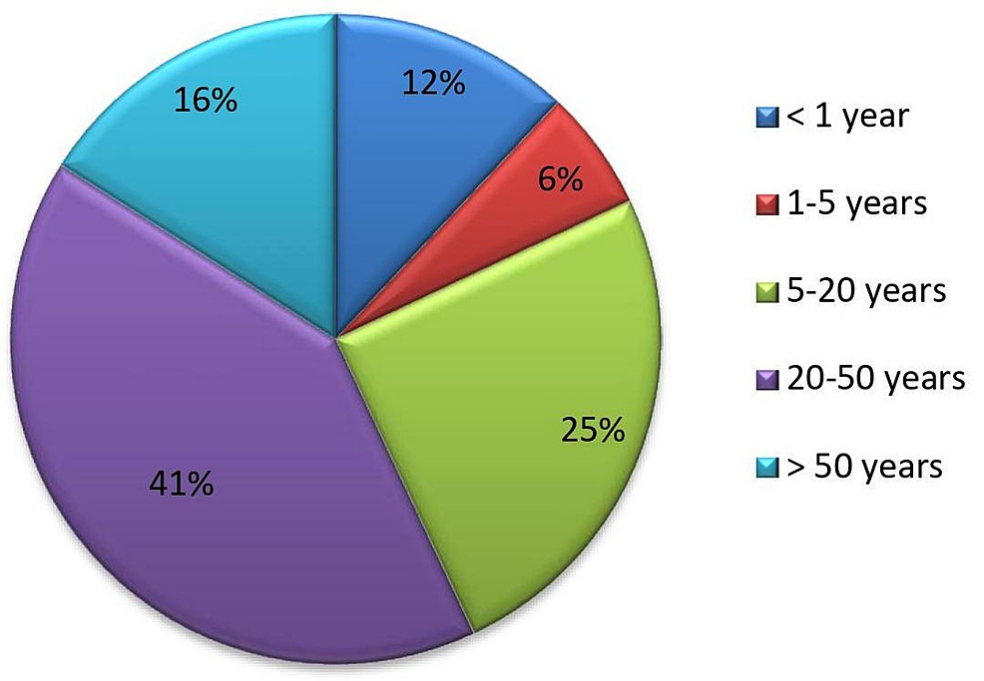
Age distribution of AUFI cases (total 721) AUFI: acute undifferentiated febrile illness.

Seven hundred twenty-one suspected cases of AUFI were studied in which 415 (57%) were males, whereas 306 (43%) were females. The majority of cases were in the age group of 20-50 years (n=295), whereas children less than one year (n=87) and from one to five years (n=44) were the least (Figure 2). Out of 721 cases, 367 were outpatients, whereas 354 were admitted to the hospital for their management. EBV VCA IgM antibodies detection assay was done for these 721 cases, where 117 samples were positive and 604 were negative. Based on the result of EBV VCA IgM, around 16% of the suspected AUFI cases (117/721) could be attributed to EBV infection. All these 117 samples were tested for EBV EBNA IgG antibodies in which 88 were positive and 29 were negative. Twenty-nine out of 721 cases (4%) were positive for VCA IgM and negative for EBNA IgG which can be categorized as primary acute EBV infection. Both EBNA IgG and VCA IgM were positive in around 88 cases which can be regarded as either EBV recovery or reactivation. The results are as tabulated in Table 1.

**Table 1:** The results of the assays performed and their clinical interpretation AUFI: acute undifferentiated febrile illness, EBV: Epstein-Barr virus, VCA: viral capsid antigen, EBNA: Epstein-Barr nuclear antigen.

No of samples	Assay performed	Reactive (number and percentage)	Nonreactive (number and percentage)
721 (AUFI cases)	EBV VCA IgM	117 & 16% (EBV infection)	604 & 84% (no EBV infection)
117 (EBV VCA IgM positive cases)	EBV EBNA IgG	88 & 96% (EBV recovery or reactivation)	29 & 4% (acute EBV infection)

## Discussion

EBV is a common viral disease especially in children and adolescents, and its symptoms resemble other febrile illnesses. Patients presenting with fever, sore throat, headache, rhinorrhea, chills and joint aches were tested for common causes of AUFI. Our objective was to describe whether some of these AUFI cases could be ascribed to EBV infection. Common modalities of EBV diagnosis include serological and molecular assays. The spectrum of serological assays for EBV includes nonspecific tests for the demonstration of heterophile antibodies (the monospot test), as well as EBV-specific assays that use different antigens with various interpretation criteria. With regards to the monospot test, there are multiple interfering factors which contribute to increased false negative results, decreasing the sensitivity and specificity as well. It has been described that in children less than four years, the sensitivity ranges between 27% and 76% [15-16]. Thus, EBV-specific antibody testing is recommended for the diagnosis of EBV [17]. We have used enzyme-linked immunosorbent assay for the detection of IgM antibodies against EBV VCA and enzyme-linked fluorescent assay for the detection of IgG antibodies against EBNA. According to Jenson, the detection of VCA IgM antibody is the most specific assay for the diagnosis of acute EBV infection [18]. The absence of EBNA-1 IgG indicates an acute infection, and their presence indicates past infection as per Sener et al. [19].

Most of our patients were adults of age group 20-50 years which is similar to a study by Grotto et al. in Israel [20], whereas Balasubramanian et al. [21] reported maximum incidence in the one to five years age group from Southern India. Other series from Turkey [22], Korea [23] and Taiwan [24] have shown similar trends in age wise incidence. The morbidity of primary EBV infection increases in patients more than 40 years of age. They may have protracted fever and serious hepatic involvement [25-26].

No gender preponderance was seen in our study as equivalent number of males and females were affected, but Son and Shin [23] from Korea noted an increasing male preponderance. No notable difference is seen in the number of patients from both outpatient and inpatient departments. The major concern in EBV disease is the detection of a primary/past or no infection [14]. Although the EBV genome codes for several nonstructural and structural genes, those encoding the EBNAs, the viral capsid antigens (VCAs) and the early antigens (EAs) are commonly detected. Therefore, the serological assays can distinguish the clinical forms of EBV [27]. Based on the outcome of our serological tests, we can categorize the AUFI cases into primary acute EBV infection and EBV recovery/reactivation.

Although the instinct is to utilise polymerase chain reaction (PCR) as a first-line test, it is documented that serology is more sensitive and specific than PCR to diagnose acute EBV infection. According to She et al., EBV DNA was demonstrated in only 70% of serology-proven acute EBV infection. Moreover, EBV reactivation cannot be detected even with highly sensitive PCR assay [28].

In our study, around 16% of the AUFI (117/721) cases could be attributed to EBV infection and around 4% (positive for VCA IgM and negative for EBNA IgG) of AUFI cases can be attributed to primary acute EBV infection. Masakhwe et al. reported that 29% of AUFI to be caused by EBV in Kenya [29].

## Conclusions

The early primary EBV infection presents with mild, nonspecific illness or silent seroconversion. Classical infectious mononucleosis syndrome with typical clinical features is rare in India. Our study should help to raise awareness regarding the possibility of EBV infection particularly in AUFI cases who do not meet the typical presentation of fever, lymphadenopathy and sore throat. Appropriate awareness about EBV infection among clinicians can reduce diagnostic confusion with more ominous diseases like malignancy which it can closely mimic. A high index of suspicion and timely diagnosis will definitely help clinicians to avoid a battery of investigations and misuse of antibiotics in cases of AUFI.
